# The Fantastic Voyage of the Trypanosome: A Protean Micromachine Perfected during 500 Million Years of Engineering

**DOI:** 10.3390/mi9020063

**Published:** 2018-02-02

**Authors:** Timothy Krüger, Markus Engstler

**Affiliations:** Department of Cell and Developmental Biology, University of Würzburg, Biocentre, Am Hubland, 97074 Würzburg, Germany; tkrueger@biozentrum.uni-wuerzburg.de

**Keywords:** trypanosoma, microswimmer, parasite, flagellate, microenvironment, tsetse, cellular waveform, microbot, trypanobot

## Abstract

The human body is constantly attacked by pathogens. Various lines of defence have evolved, among which the immune system is principal. In contrast to most pathogens, the African trypanosomes thrive freely in the blood circulation, where they escape immune destruction by antigenic variation and incessant motility. These unicellular parasites are flagellate microswimmers that also withstand the harsh mechanical forces prevailing in the bloodstream. They undergo complex developmental cycles in the bloodstream and organs of the mammalian host, as well as the disease-transmitting tsetse fly. Each life cycle stage has been shaped by evolution for manoeuvring in distinct microenvironments. Here, we introduce trypanosomes as blueprints for nature-inspired design of trypanobots, micromachines that, in the future, could explore the human body without affecting its physiology. We review cell biological and biophysical aspects of trypanosome motion. While this could provide a basis for the engineering of microbots, their actuation and control still appear more like fiction than science. Here, we discuss potentials and challenges of trypanosome-inspired microswimmer robots.

## 1. Introduction

The conception of engineering tiny machines that could travel inside a living body and function as nano-medical devices therein, is attributed to Richard Feynman, who, in his visionary talk, quoted the idea of producing miniature devices that could reach any part of the body, there to perform delicate surgical tasks [1]. A couple of years later this vision was realised, albeit only on the silver screen, when a miniature submarine was injected into the patient together with its crew, in order to remove a blood clot in the film “Fantastic Voyage”. Shrinking such a manoeuvrable vehicle to micrometre dimensions, with or without human pilots, remains science fiction, but the development and operation of micro-engineered devices that could eventually swim through a living body, are currently being pursued in an increasingly active field of research.

Micromachines, typically up to several tens of micrometres in size, are being developed in the field of micro- or nanorobotics. The aim is for the devices to act at the nanoscale, i.e., on the molecular level. Accordingly, medical micro- or nanobots would be micromachines powered by nanoscale motor mechanisms and perform in the delivery of molecules or manipulations of molecular and cellular structures in specific regions of the body [2,3,4,5,6]. The first major challenges for realisation of this concept lie in the creation of mechanisms for propulsion and navigation of the machines in the bloodstream and in tissues, i.e., the engineering of controllable microswimmers [7,8,9,10].

**Figure 1:**
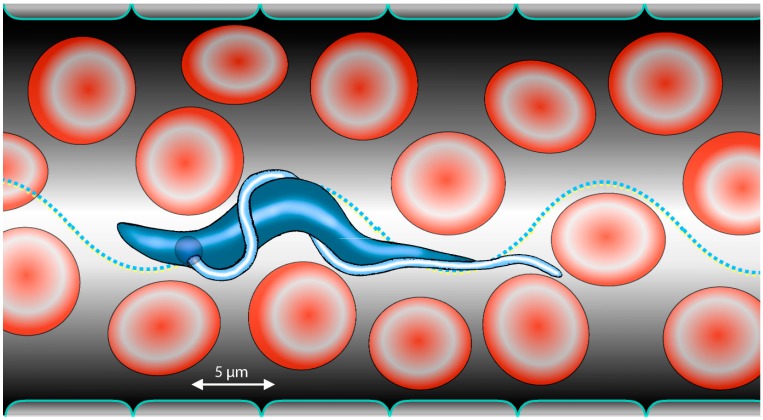
Motion of trypanosomes in the blood of their mammalian host is adapted to the crowded microenvironment. The presence of obstacles in the form of blood cells accelerates speed and improves directionality of parasite swimming.

Several artificial microswimmer types have been designed and developed, and some biological counterparts have been analysed and manipulated in order to perform controlled navigation and delivery tasks in vitro and in vivo [11]. The implemented natural swimming models include bacteria with helical, rotating flagella [12,13], eukaryotic ciliates [14,15,16], as well as flagellates [17,18]. In this review, we would like to introduce a microswimmer that has yet to be considered as a suitable organism for nature-inspired microrobotic design, the trypanosome. This eukaryotic, flagellate cell has uniquely adapted to swimming in the bloodstream (Figure 1) and in tissue spaces of vertebrates, including humans, as it has co-evolved in these hosts as an extracellular parasite during a time span of about 500 million years [19]. Therefore, we can assume that the design of this extremely versatile and successful parasite is exemplary for any “submarine” that is meant not only to reach any part of the body via the circulatory system, but also to further navigate in distinct tissues and organs. The very effective propulsion and manoeuvring capabilities in considerably varying physical environments, makes for the perfect candidate to inspire medical microrobot design.

## 2. Trypanosomes—Cosmopolitan Parasites

The trypanosomes, which are exclusively parasitic protists, are divided into two clades, the Latin American *Trypanosoma cruzi*, which causes Chagas disease, and the African trypanosomes, which cause human sleeping sickness and several animal trypanosomiases. The parasites of both groups survive in the blood and tissues of vertebrates employing different strategies. Whereas *T. cruzi* invades cells and develops intracellular stages, the African trypanosomes manage to survive extracellularly throughout their life cycle. This is no mean feat, because the blood-dwelling parasites are constantly under attack by the host´s immune system. The trypanosomes have developed efficient mechanisms to escape immunological destruction and hence can be deadly, but only for certain hosts [20,21]. Actually, in a wide range of well adapted hosts, a multitude of African trypanosome species persist harmlessly, continuously switching between vertebrate hosts and invertebrate vectors. 

## 3. The Model Trypanosome

**Figure 2:**
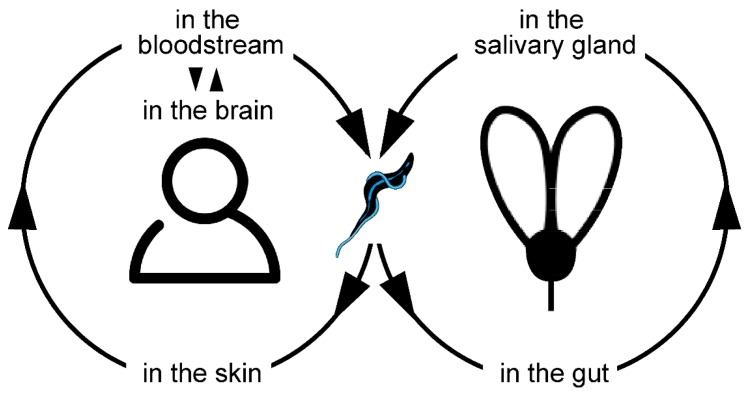
Trypanosome life cycle. The trypanosomes shuttle between the mammalian host and the insect vector. In human infections, the parasites travel from the skin to the circulation and later on, colonise the brain fluids. After uptake with the blood meal of a tsetse fly, the trypanosomes undergo a complex developmental cycle within the insect, before they are re-injected into the host.

The best studied and most versatile parasite is *Trypanosoma brucei*, of which four different subspecies are recognised that cause either human (*T. b. gambiense*, *T. b. rhodesiense*) or animal (*T. b. brucei*, *T. b. evansi*) African trypanosomiasis. The cattle pathogen *T. b. brucei* is used routinely as a model organism for molecular cell biology, genetics, parasitology and more. Fortunately, humans are resistant to infections with this parasite, as it is killed by human serum [22]. *T. b. brucei* is transmitted with the saliva of the tsetse fly during the blood meal of the biting insect (Figure 2). Within the fly, the parasite undergoes a complex developmental cycle [23,24]. Once re-injected into the mammalian host, the life cycle continues with the adaptation to blood and tissues, where the trypanosomes circulate and proliferate, awaiting retransmission to the tsetse [25,26].

During this dioxenous life cycle, trypanosomal development exhibits a whole range of distinct cell morphotypes. Some of these cell forms can easily be maintained in cell culture and can be induced in vitro to change into one another. Other morphotypes are developed in the tsetse fly vector, which can also be kept readily in the laboratory [27]. The cellular designs adopted by *T. brucei* represent a good selection of flagellate morphological classes, which are developed from a conserved cytoskeletal and membranous, eukaryotic design [28,29,30]. These include trypomastigote and various epimastigote stages, i.e., cells with an attached or a free flagellum of highly variable length. It is this programmed morphological variability of trypanosomes that make them model microswimmers. Each life cycle stage is perfectly adapted to perform special tasks in the varying micro-environments within the human and the tsetse fly. In the following, we review the current detailed knowledge of trypanosome morphotypes and their motility, with the goal in mind, to eventually understand and control functional design principles for navigation in the human body.

## 4. The Sinuous Basic Motion Pattern

Trypanosomes swim by producing a travelling wave along a single flagellum, which pushes the surrounding liquid in one direction and, thus, generates a propulsion force in the opposite direction. This type of hydrodynamic impulse is described by the resistive force theory [31]. The bending force for flagellar oscillation is produced by dynein motors in the axoneme, the conserved core of all eukaryotic flagella and cilia [32]. The propagation of the axonemal bend produces non-reciprocal waves, as required for low Reynolds number swimmers [33]. Thus, the resulting movement is equivalent to that of sperm cells, the classic flagellar microswimmer model [34]. In contrast to sperm cells though, in most trypanosome forms, the flagellum is attached in a helical manner to the greatest part of the long, spindle-shaped cell body. The travelling wave thus deforms the elastic body and the whole cell oscillates, producing a cellular waveform which determines the trajectory of the cell [35]. The directional swimming cell rotates around its longitudinal axis because of the chiral attachment of the flagellum [36,37].

A particularly significant and, in fact, unique feature of trypanosomes is their ability to produce flagellar waves from both ends. Forward movement is caused by a wave running from the free anterior (front) tip of the flagellum to its base, which is anchored in the flagellar pocket at the posterior (rear) end of the cell. The parasites can also produce waves running in the opposite direction, from the flagellar base to the tip, usually with irregular frequencies. The default mode in low-viscosity fluids is either continuous beat reversal, which leads to the equalisation of net propulsive forces, resulting in a tumbling movement of cells, or a forward movement stochastically interrupted by single reverse waves that change swimming direction. The predominant mode depends on the morphotype examined, but, importantly, it is also strongly influenced by the physical environment [27,35]. Exclusive base-to-tip beating is rare, but causes the trypanosomes to switch to backward swimming, either after experimentally inhibited forward waves or by mechanical confinement [36].

## 5. Blood Microswimmers

**Figure 3:**
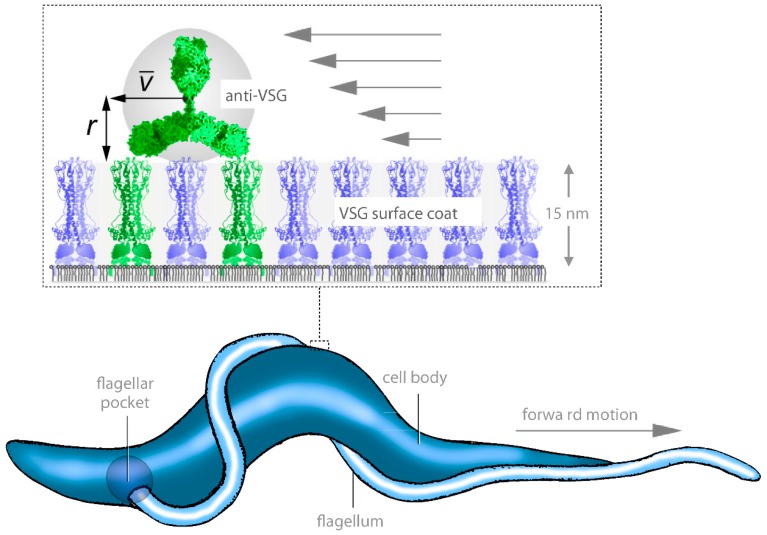
Hydrodynamic antibody clearance. The concerted action of cell motility and localised endocytosis is responsible for antibody clearance: As a single type of variant surface glycoprotein (VSG) covers the complete trypanosome cell body, the host immune response is directed specifically against this surface coat. VSGs are Glycosylphosphatidylinositol (GPI)-proteins that are specifically anchored to the outer leaflet of the plasma membrane. This lipid-modification makes VSGs highly mobile proteins; they diffuse freely and rapidly in the membrane. Antibodies that bind to VSG (green) extend above the unusually smooth surface coat and are dragged by hydrodynamic flow towards the posterior part of the cell, where the very efficient endocytosis machinery resides. The hydrodynamic drag acting on the VSG-bound antibodies originates from the constant and directional puller-type motion of the parasites.

The first example of swimming behaviour adaptation in a distinct environment is described through the analysis of the *T. b. brucei* bloodstream form (BSF). This cruiser of the mammalian bloodstream evades the immune system by periodically changing the molecular fabric of its cell surface coat. The trypanosome coat is made of a single type of variant surface glycoproteins (VSGs). In a process called antigenic variation, the trypanosomes stochastically exchange one VSG coat for another, picking from a repertoire of hundreds [38]. Cells that do not change their surface coat are recognised by the host´s immune system, and will sooner or later have antibodies against their VSG bound to their coat. At this point an important benefit of directional swimming ability comes into effect, as the trypanosomes escape immune destruction by hydrodynamically removing the bound antibodies. In order to achieve this, they need to swim forwards with sufficient speed and let the drag force of the surrounding fluid pull the exposed antibodies to the rear end of the cell (Figure 3). Here they will automatically accumulate in the flagellar pocket, the only entrance into the cell via endocytosis and, thus, be destroyed [39]. This mechanism could protect the parasite from immune clearance as long as the antibody titer is low, which is the case in early infection stages.

Although the trypanosomes have not yet been recorded live in the mammalian bloodstream, it has been shown that under in vitro conditions, the parasites will reach the required speeds when they are given a mechanical impetus to swim directionally, either by raising the surrounding fluid´s viscosity, or by providing regularly spaced pillar arrays, which simulate the mechanical situation in blood vessels densely packed with erythrocytes. On average, the human erythrocytes, which have a diameter of around 7 µm, are spaced about 4 µm apart. The trypanosomes can surge though such spaces, using the pillars (blood cells) as bumpers for threading the deflecting flagellum through effectively narrow channels [36].

## 6. Location Matters

These early results implied that the speed and directionality of trypanosomes can be controlled by purely mechanical means. This is also very important for the movement in tissue spaces. Although it is unclear how the parasites passage from blood vessels to tissues and vice versa, it is clear that several organs are habitable niches for the parasites. First of all they transit the skin tissues they are deposited in by the mouth parts of the insect that transmitted them [26,40]. Then they colonise the lymph system, adipose tissue [41] and, finally, the central nervous system, after crossing the blood brain barrier in the last stages of disease [42]. Examination of trypanosome life and motility in these habitats has only begun, but it has become clear that the trypanosome cell design and swimming mechanism are perfectly suited for manoeuvring through spaces with varying physical properties. The preferred niches can even be correlated with the morphological characteristics of different trypanosome species [35]. All morphotypes are based on one cytological principle design, but with variations thereof, they are able to navigate several ranges of viscosity, as well as confined regions, tightly packed with dense materials, due to their flexible body and screw-like, bidirectional motion. Surprisingly, nowhere do these abilities become clearer than in analyses of the developmental cycle in the tsetse fly.

## 7. The Multifarious Tsetse System

As introduced above, *T. brucei* parasites undergo a series of developmental stages in the organs of the tsetse fly, in which they live for weeks in between blood meals taken from successive mammalian hosts. The first trypanosome stages in the fly, called procyclic forms (PCF), are distinct from the BSF cells, but the general morphology and flagellar propulsion are very similar. The insect stages of *T. brucei* have been examined in detail and analysed in almost every possible motility status. The cells swim efficiently in blood and in low viscosity fluid, but also in and on epithelial tissues, as well as through channels, crevices and on surfaces of a strongly convoluted fly structure called the peritrophic matrix. The trypanosome-fly system thus provides a convenient and exhaustive test ground for all kinds of microswimmers in virtually any degree of confinement [27]. What is clear from observations in fly tissue, but also from skin tissue culture, is that the bidirectional modus of flagellar wave propulsion allows the trypanosomes to efficiently explore any magnitude of topological space in a body system, even down to tight networks of, say, collagen in the epidermis.

In the course of development in the fly, trypanosomes produce significant alterations to the above described trypomastigote morphotype. Asymmetric divisions occur to produce small and less motile cells on one hand and cells with long, free flagella, which thus resemble sperm cells, on the other. The motility must play an important role, as long flagella make sense for cells that need to travel long distances quickly through fluid spaces, whereas relatively immotile cells go on to attach to epithelia, in order to develop further [23,24,43,44,45]. These processes are understudied, but the enormous potential harboured by the variations of a single cell design, for a broad palette of functions inside of a body, be it arthropod, domestic mammal or human, seem obvious to the observer interested in nature-inspired microrobotic engineering.

**Figure 4:**
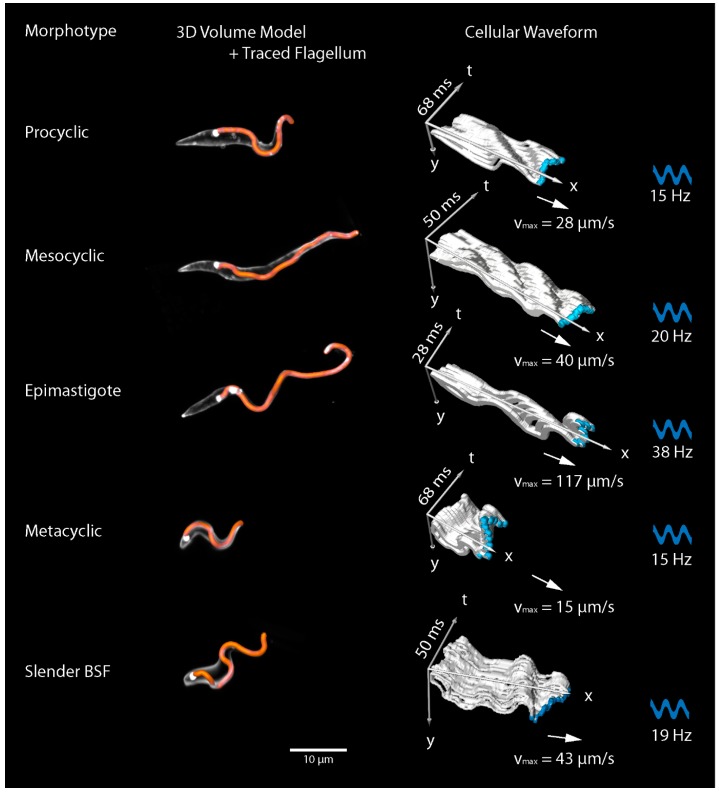
Selected *T. b. brucei* morphotypes and dynamic cellular waveforms. Major developmental stages are shown, representing pivotal transitions in the parasite´s life cycle. The development from procyclic cells to metacyclic cells takes place in the tsetse fly. Metacyclic cells are injected into mammals and transform into the blood dwelling forms. The left column depicts 3D volume models from fluorescently surface stained, fixed cells. The cell membrane signal allows to trace the course of the attached flagellum around the cell body. These data assess the chirality of the microswimmers and allow the interpretation of their basic rotational motility mode. The morphology correlates with the characteristic swimming behaviours of the different stages. The right column represents traces from high speed recordings of forward swimming cells of the corresponding morphotype. The two-dimensional silhouettes of the cells from successive frames (4 ms resolution) are combined to produce a three-dimensional visualisation of movement along the time axis, as described in detail in [35]. The resulting kymograph reveals the shape of the travelling wave produced by each flagellar beat. Beat frequency, wave amplitude, and maximal velocity, which are measured directly from the original data, are characteristic for specific cell stages, and in combination with their characteristic elasticity, determine the motile capabilities in various surroundings. Figure modified from [27,35].

Importantly, current efforts reveal possibilities to quantitatively analyse the complex variety of microswimmer designs with high spatiotemporal resolution. The complex three-dimensional movement of flagella and cell bodies of the trypanosome morphotypes were recorded in single cells, as well as large cohorts of collectively swimming cells. The variety of three-dimensional shapes and the elasticity of the cells oscillating with the attached flagella can be described with kymographic representations of the dynamic cellular waveform (Figure 4) [27]. The results allow the quantification of single parameters, such as flagellar beat frequency, amplitude of oscillation, flagellar wave speed, deformation of the cell body and the resulting velocity and three-dimensional trajectories of the parasites.

These details are prerequisite for the mathematical models of microswimmers, which, in turn, are essential for the simulation of micromachine designs. The full-scale realistic numerical simulation of a swimming trypanosome in variable surrounding fluids, represents an important step towards understanding and reproducing a functional microswimmer [37]. Live high-speed 4D-microscopy of these cells is currently not feasible [46], but with the simulation approaches, especially by multi-particle collision dynamics (MPCD), we are able to predict details of morphological design that are not yet observable. The simulated motility behaviour of these designs can be checked for plausibility by in vivo analysis and the simulations refined accordingly. Beyond that, the numerical designs can be adjusted in such a way as to produce entirely experimental microswimmers, which would assist in elucidating the functional significance of evolutionary micromachine engineering.

To this effect, variations of trypanosome morphotypes are also observable across species. *T. vivax*, *T. congolense*, and *T. b. evansi* have adapted to preferentially different niches inside the bodies of different animals [47,48,49,50]. The BSF forms of these species retain the ability to proliferate in the circulation, as well as in tissues, albeit they exhibit marked differences in morphology and motility. Noteworthy is also, that they are often found in mixed infections [51,52], therefore, the morphotypes might be able to share a common host, simply by virtue of differing in motility behaviour and, therefore, in preferential microhabitats. In other words, we are presented with an evolutionarily-established in vivo sorting mechanism, based on variations of a single cell type.

## 8. Towards Bioinspired Trypanobots

While we shed light onto several aspects of trypanosome microswimmer design during development and evolution, we realise how useful such a versatile micromachine would be as a medical micro- or nanorobot. As soon as we understand how certain parasite species and subspecies adapt their morphotypes to specific microhabitats in the host body, we could think of using these concepts in order to create ‘trypanobots’, micromachines whose design is tuneable to their intended deployment destination in the body. The evident navigational properties of the elastic trypanosome cell body with the attached bi-directional flagellum, could make such a micromachine more than a single use delivery tool. Instead, the trypanobot could return to the bloodstream, after reaching any location in the body. Understanding the physical principles the trypanosomes exploit, in order to efficiently switch between navigating fluids and tissues, gives us the opportunity to learn how to engineer veritable ´body shuttles´. The screw-like movement of the whole swimming body with the attached flagellum is apparently nature´s state of the art design principle.

What is needed to approach this, admittedly ambitious, goal of reverse engineering? We hypothesise that Louis Sullivan’s maxim “form follows function” [53] also applies to trypanosome microswimmers, i.e., all parasite morphotypes have been shaped by evolution for the ability to effectively manoeuvre in distinct environments. Detailed blueprints of the 3D shape of all trypanosome life cycle stages are available, as are high-resolution measurements of flagellar beat pattern and parasite velocities. An important parameter, however, is still missing, the bending stiffness of the parasites. This is a problem, as the elasticity of the trypanosome cell body is a key-feature of the varying cellular waveforms. In other words, we need to know the material properties of the trypanosome cell before we can design nature-inspired trypanobots. Measuring the elastic modulus of trypanosomes is aggravated by their spindle-like shape. The use of optical tweezers has provided an estimate of trypanosome force generation and, hence, an indirect measure of the bending properties [54]. A much more precise and high-resolution determination of the cell elasticity exploits a particular feature of the trypanosome cell architecture, the microtubule cytoskeleton that extends just beneath the cell surface throughout the entire parasite [55]. This microtubule corset determines the stiffness of the trypanosome. The use of serial electron tomography allows detailing the complete cytoskeletal array and, thus, provides a highly-precise elasticity map of a given trypanosome morphotype. This completes the parameters required for designing prototypic trypanobots. For the actual fabrication could be attempted using two-photon polymerisation, which is ideally suited for producing objects in the size range of trypanosomes (20–50 µm), with a resolution of 200–500 nm [56,57]. While building the trypanobot’s chassis appears rather straightforward, implementing the engine and actuation certainly is not. Ideally, a reversibly- and sinusoidally-beating filamentous actuator would be attached in a half-turn around the trypanobot.

The trypanosomal design can be seen as an extension of the spermatozoan principle. The common propulsion system of an axoneme producing travelling waves, should principally allow for the application of any force producing and motility control technology developed for the use in sperm or spermbot motility research to trypanosomes. Once a propelled trypanobot is developed, the control mechanism should be able to produced bidirectional waves. As the axonemal control mechanisms for these kind of movements are still largely unknown, the research of trypanosome inspired propulsion systems would advance basic knowledge, as well as micromachine development.

The concept of integrating a robust, deformable cell body into this kind of swimming device is interesting, as it conveniently ensures the unperturbed locomotion of a compact micro-transporter. The structural internal cellular design of trypanosomes is decisive in that respect. It consists of a stable corset of microtubules, which is nevertheless quite bendable. As stated above, the elasticity of trypanosomes has not been quantified, but the degree of deformation in fly tissues, for instance, demonstrates a high degree of flexibility [27]. A vehicular architecture fabricated from such a tubular frame would be desirable for a micro transporter. Think of a robust soft-hull submarine with the agility of a snake.

Trypanosomal design offers many more specifications that could be exploited for artificial microbots. Imagine the capability to construct a vehicle with a semi-fluid surface coat, like the cell membrane, which is able to bind specific molecules that can be dragged into a pocket and consequently internalised, simply by fast, directional swimming. This gimmick would be invaluable either for collecting transport cargo inside the machine, or in order to degrade material. The latter function would enable a handy micro-hoover, scavenging the body for specific superfluous molecules or particles. Theoretical micromachines of this kind have been described with a high degree of technical sophistication (e.g., microbivores, [58]). Nature-inspired mechanisms from trypanosomes might aid in simplifying future engineering processes.

Attachment sites on a uniform surface coat, like the trypanosome´s VSG mantle, could specifically and reversibly be loaded with cargo or variable hull structures for different passages. An attached, but compartmentalised, appendage, like the flagellum, provides an opportunity to attach specific structures, such as sensory equipment. As exemplified by the evolutionary development of sensory cilia [59], sensors or receptors could be located and concentrated in defined numbers, independent of the main cell body. Such sensors could be exposed while swimming, or in direct contact with tissue if the cell is enabled to attach to it via the flagellum.

The analysis of trypanosome morphotype variation allows us to explore the capabilities of microswimmers with diverse propulsion systems and attached cell bodies. This means that we have natural examples of deconstructed micromachines. For a large amount of modified machines we can produce, there will likely exist a corresponding parasite. Take the variable length of the free flagellum, which could be modified in order to customise not only motility behaviour, but also the available sensor domain. Now swap the sensors with micro-tools that can be moved with the flagellum and we might be able to create the surgeon that was “swallowed” [1]. If you somehow manage to build micro cameras to add to this design, you have another requirement for operations inside the body, your trypanobot could not only feel its way through the body but also see where the fantastic voyage is going.
